# Supplementary material for: Unconventional correlated insulator in CrOCl-interfaced Bernal bilayer graphene
Source: Nat Commun. 2023 Apr 14;14:2136. doi: 10.1038/s41467-023-37769-2 (PMC10104821; doi:10.1038/s41467-023-37769-2)
Supplement: Supplementary file 1 — Supplementary Information [file 41467_2023_37769_MOESM1_ESM.pdf]

Supplementary Information for

# Unconventional correlated insulator in CrOCl-interfaced Bernal bilayer graphene

Yang, Gao, Wang, and Zhang, *et al.*

**Outlines**


**1.** The work flow of sample fabrication progress.

**2.** Morphology and atomic resolution of the cross section of a typical sample.

**3.** Comparison of gapped state between BLG/CrOCl and h-BN/BLG/h-BN.

**4.** Reproducibility of the exotic insulating phase in CrOCl-interfaced BLG.

**5.** Channel resistances of dual gated BLG/CrOCl under magnetic fields.

**6.** Supplementary Note 1. The electrostatic model of the h-BN/Bilayer Graphene (BLG)/CrOCl.

**7.** Temperature dependence of dual gated $R_{xx}$ in BLG/CrOCl.

**8.** Linear dependence of switching voltage from insulating to metallic state.

**9.** Logic devices based on correlated insulator in BLG/CrOCl heterosystem.

### 1. The work flow of sample fabrication progress.

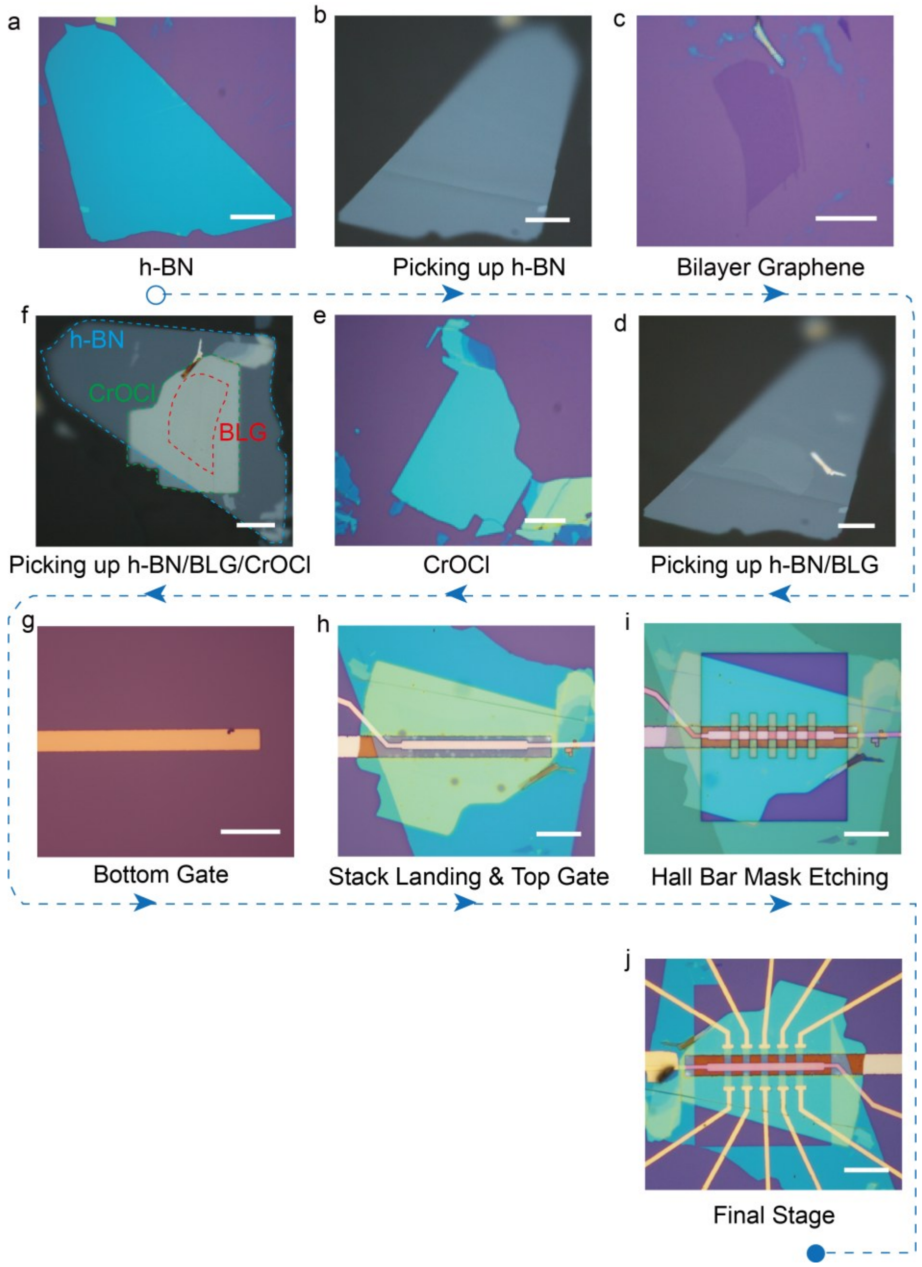


**Supplementary Figure 1. Sample fabrication process.** (a)-(j) show the flow of a typical fabrication process of the samples. Bernal-stacked bilayer graphene (BLG) as well as thin CrOCl flakes (10-20 nm in thickness) and encapsulating hexagonal boron nitride (h-BN) flakes (10-20 nm in thickness) were exfoliated from high-quality bulk crystals and stacked in ambient condition using the polymer stamp assisted transfer method.[1] Electrodes with edge-contacts to Hall bars of the such vertically assembled van der Waals (vdW) heterostructures were patterned using standard electron beam lithography. A dual gate configuration is used to capacitively induce carriers $n_{tot}$ and to define an effective vertical displacement field $D_{eff}$ from both top and bottom gates. The recipe of $O_2/CHF_3$ for etching h-BN has a negligible etching rate with respect to CrOCl, as indicated in (i) h-BN/BLG has been etched away while CrOCl is almost not affected by the etching. Scale bars in these images are 10 μm.

## 2. Morphology and atomic resolution of the cross section of a typical sample.

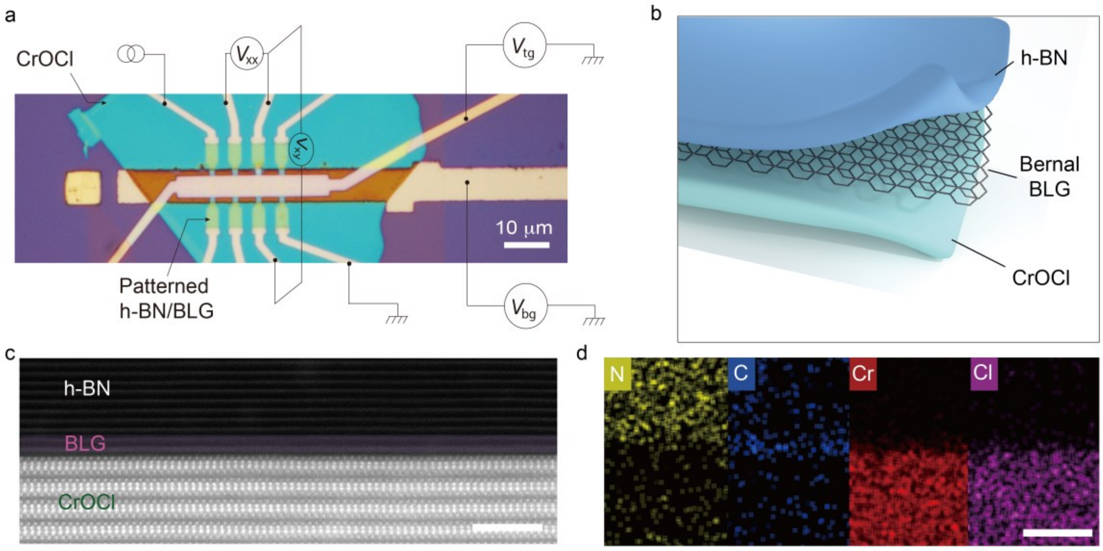


**Supplementary Figure 2. Cross-section of a typical sample fabricated** (a) Optical micrograph of a typical sample, with the aspect ratio of the measured channel close to 1 in Fig. 1 in the main text. (b) Cartoon view of the h-BN/BLG/CrOCl samples described in (a). (c) Atomic resolution of the cross section of a typical heterostructure can be seen in the high-angle annular dark-field scanning transmission electron microscopy (HADDF-STEM) image, with the corresponding electron dispersive spectroscopy (EDS) mapping shown in (d).

## 3. Comparison of gapped state between BLG/CrOCl and h-BN/BLG/h-BN.

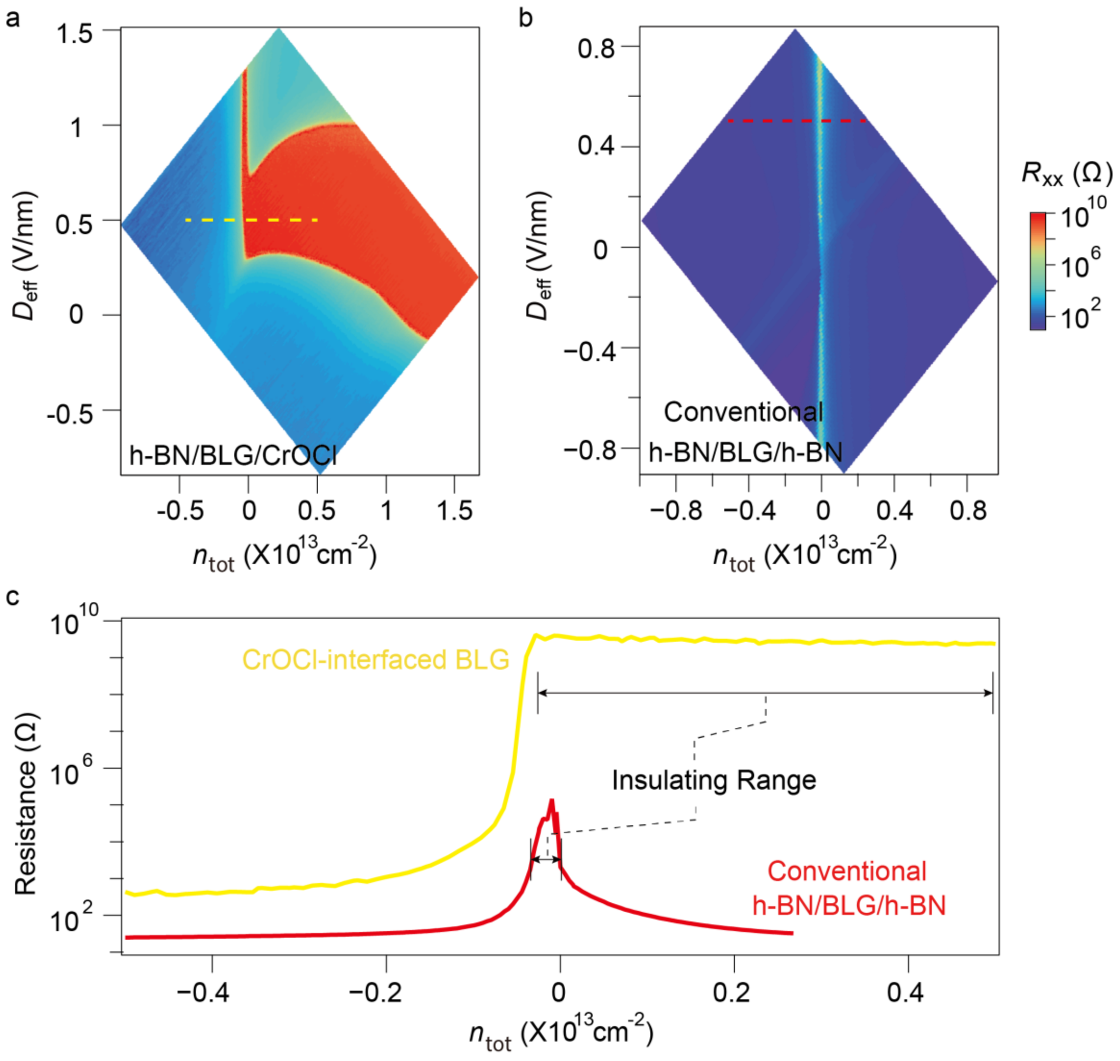


**Supplementary Figure 3. Comparison between CrOCl-interfaced BLG and the conventional h-BN sandwiched case.** (a) Same data in Fig. 1e in the main text plotted in the $n_{tot}$-$D_{eff}$ space. (b) $R_{xx}$ obtained in a control sample of conventional h-BN/BLG/h-BN device. (c) Line profiles of the exotic insulating phase in CrOCl-proximitized BLG and conventional h-BN sandwiched BLG, plotted in the similar range of $n_{tot}$ and $D_{eff}$. It is seen that GΩ resistance can be found in much wider effective doping range in the BLG/CrOCl system.

| System | Maximum Resistance (Ω) | Effective Doping Range of the Insulating State ($\times 10^{12}$ $cm^{-2}$) | Displacement Field at Maximum Resistance (V/nm) | Ref. |
|---|---|---|---|---|
| h-BN/BLG/h-BN | $0.7\times 10^{6}$ | ~ 0.5 | N/A | [2] |
| $Al_2O_3$/BLG/$SiO_2$ | $5.0\times 10^{8}$ | ~ 2.0 | -2.3 | [3] |
| Suspended BLG | $1\times 10^{6}$ | ~ 0.03 | N/A | [4] |
| h-BN/BLG/h-BN in Corbino Geometry | $0.7\times 10^{7}$ | < 0.5 | ± 0.6 | [5] |
| Suspended BLG | $1\times 10^{7}$ | ~ 0.4 | 0.09 | [6] |
| h-BN/BLG/CrOCl | $5.0\times 10^{9}$ | > 10.0 | [0, 1] | This Work |

**Supplementary Table 1. Summarization of characteristic parameters for different BLG samples reported.[2-6]**

## 4. Reproducibility of the exotic insulating phase in CrOCl-interfaced BLG.

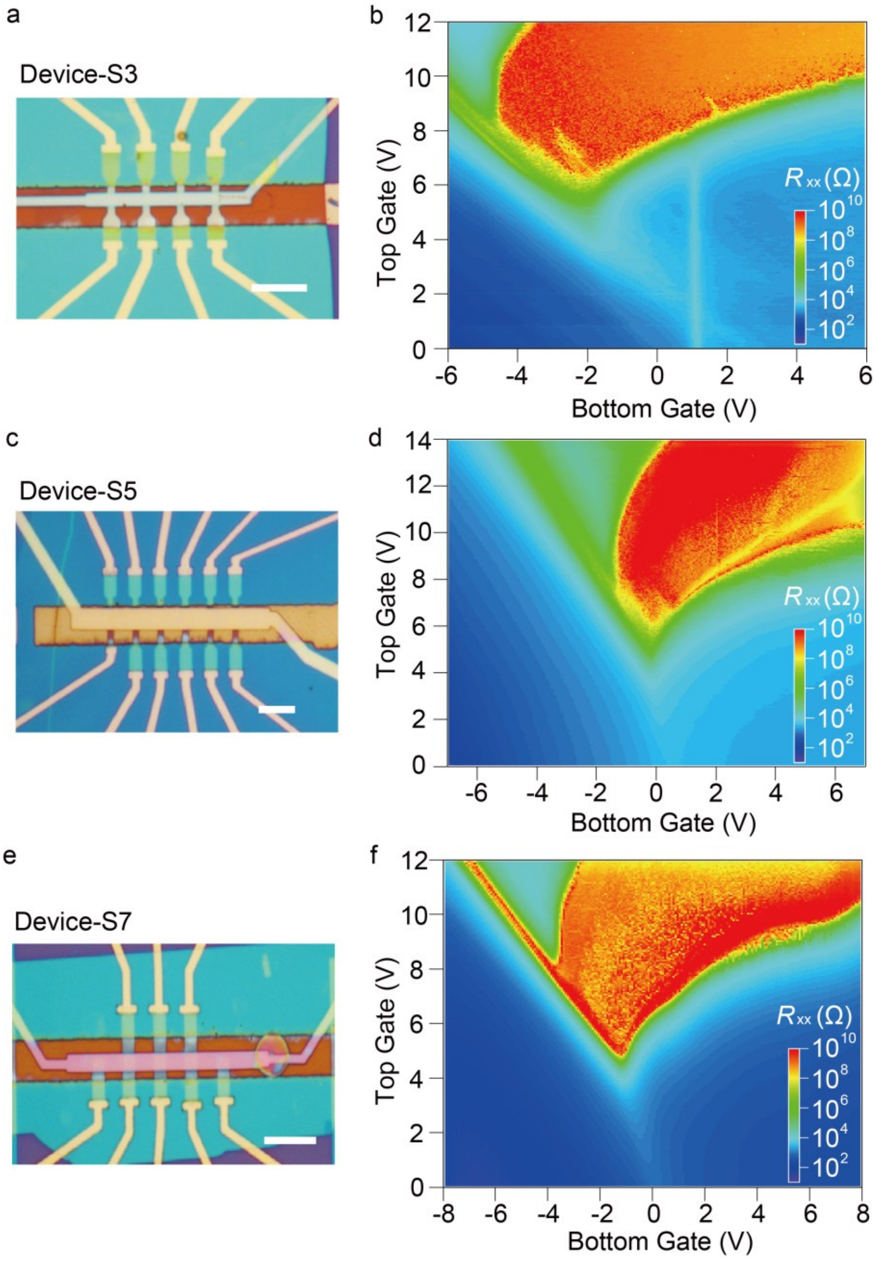


**Supplementary Figure 4. Reproducibility of the observed insulating phase in BLG/CrOCl.** Typical devices of the h-BN/BLG/CrOCl heterostructures measured at temperature of 1.5 K, with their optical image and channel resistances $R_{xx}$ plotted side by side, from (a) to (f), respectively. It is seen that the insulating phase in Fig. 1e in the main text is reproducible in multiple samples tested.

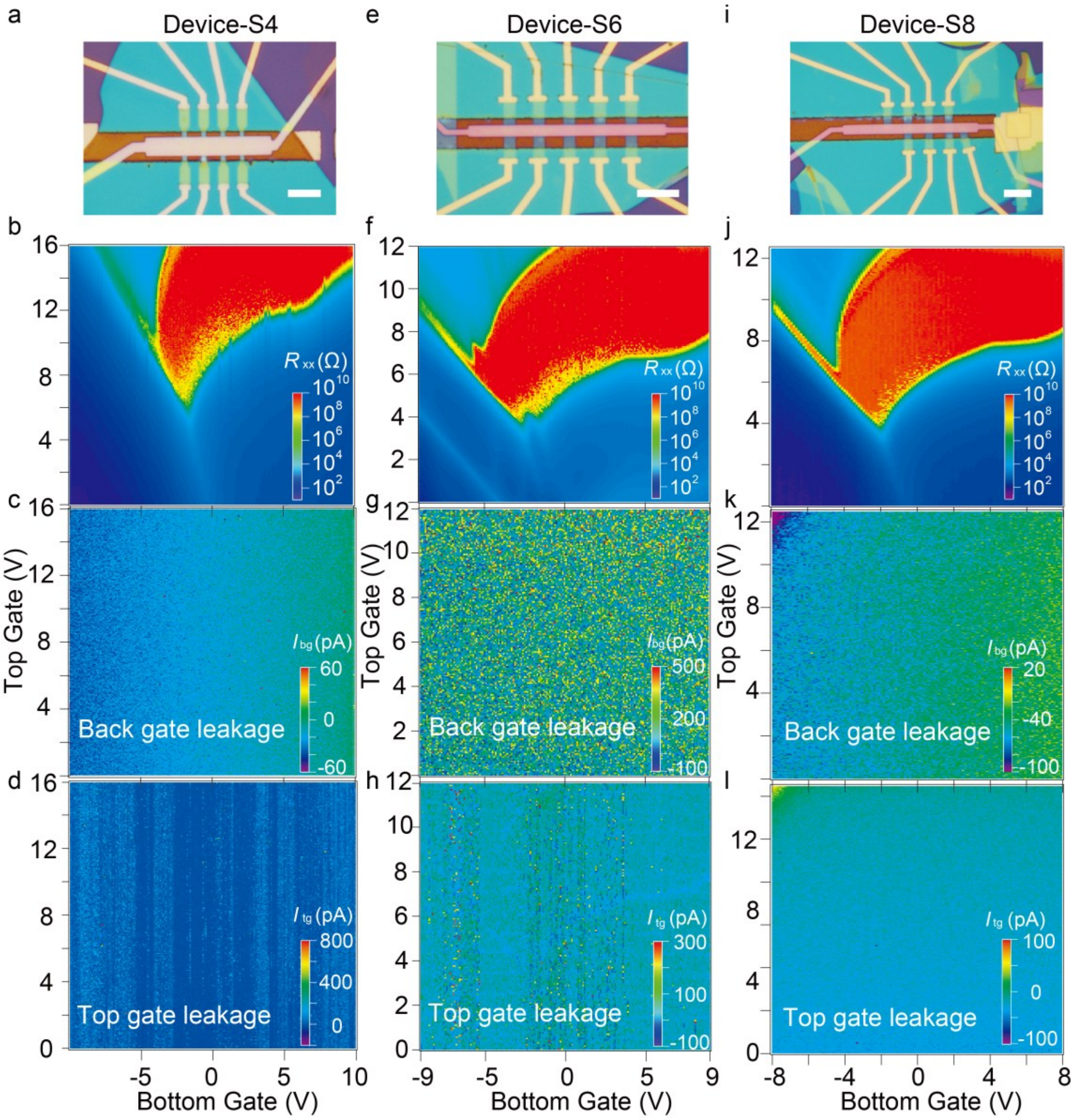


**Supplementary Figure 5. Negligible gate leakage currents in the devices studied.** By examining the gate leakage currents in both top and bottom gates, we found no leakage currents in the samples (a)-(d) device S-4, (e)-(h) device-S6, and (i)-(l) device-S8. Few layered CrOCl is proven to be a good dielectric material at low temperatures. These data were measured at $T = 1.5$ K.

## 5. Channel resistances of dual gated BLG/CrOCl under magnetic fields.

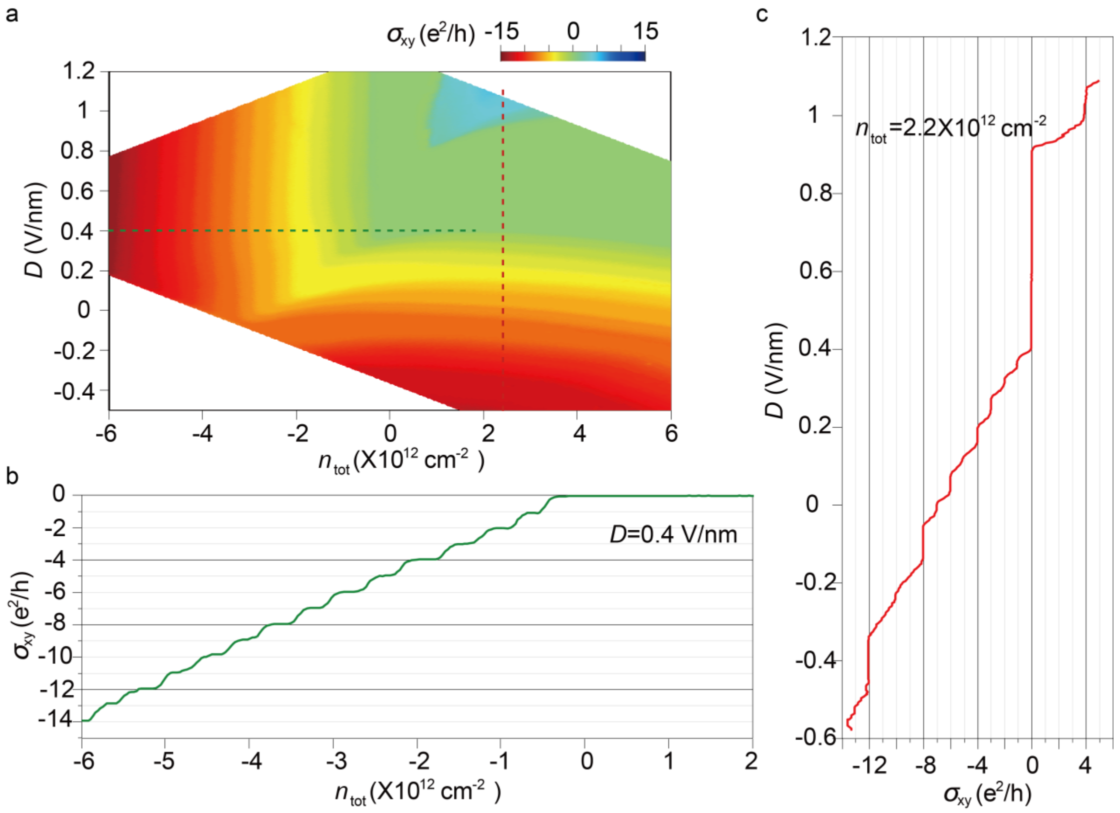


**Supplementary Figure 6. Landau quantizations (in $\sigma_{xy}$) in a typical bilayer graphene (BLG)/CrOCl sample.** (a) Color map of $\sigma_{xy}=R_{xy}/(R_{xx}^2+R_{xy}^2)$ plotted in the parameter space of $n_{tot}$ and $D$, at $T$ = 1.5 K and $B$ = 12 T. (b) and (c) are line profiles of $\sigma_{xy}$, obtained at $D_{eff}$=0.4 V/nm and $n_{tot}$ = $2.2\times10^{12}$ cm$^{-2}$, respectively. In the conventional phase, full degeneracy lifting was seen, with integer filling fractions of $\nu$ = 0, -1, -2, -3, -4, …, all well defined in the quantum Hall plateaus in $\sigma_{xy}$. In the interfacial coupling phase, electron doping can be realized, with $\nu$ = + 4 visible. In order to extract the $n_0$ and $D_0$ (defined in the main text), we define the $n_{tot}$=0 using the LL of $\nu$ = 0 outside the unusual gap, and hence $n_0$ can be obtained. Since $D$ is a relative value, we chose $D_0$ at the section of $n_{tot}$=0 line with $V_{bg}$=0, for simplicity.

BLG has valley (+,-), spin(↑,↓), plus the orbital (0,1, i.e., layer polarization), a total of 8-fold 'degeneracy' in the lowest Landau level. Experimentally, full degeneracy lifting has been observed in previous reports.[2,7] It is seen that all integer filling fraction of -1, -2, -3, -4,… can be found in those samples in the clean limit, which is similar to our results in Fig. 2 in the main text. The difference is, in the conventional BLG cases, energy levels in the zero energy Landau level (ZLL) are crossing in the space of $D$ and $n$ at a certain magnetic field $B$. This is not seen in our CrOCl-interfaced BLG samples, we attribute it to a high $D_{eff}$ range where crossing were not seen. While in the conventional case the interlayer potential $u$ has to go

through zero and crossings are inevitable at low energies. At this stage, we would tend to think that such valley symmetry broken phenomenon is unlikely related to the observed correlated insulator behavior.

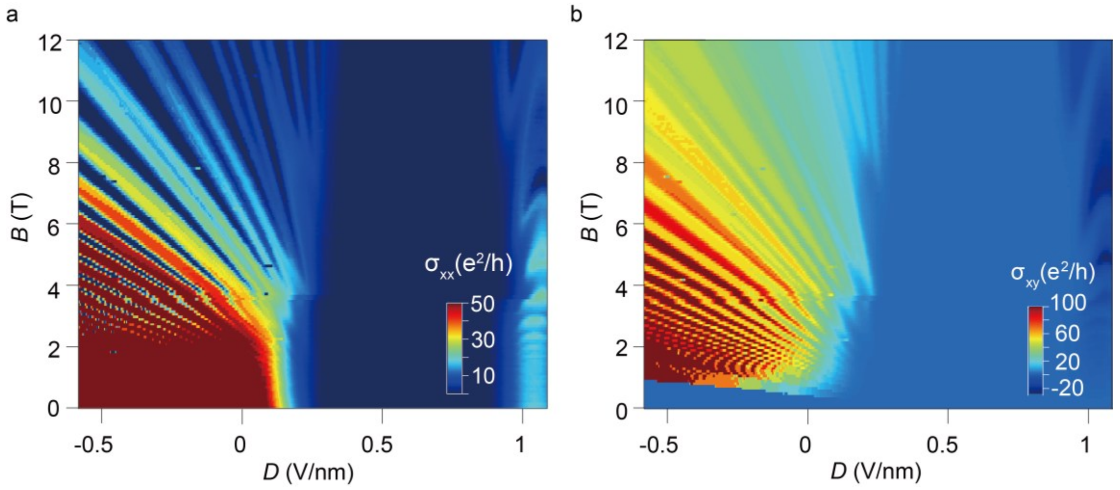


**Supplementary Figure 7**. **Landau fan of a typical BLG/CrOCl device scanned along with different $D_{eff}$ at a fixed $n_{tot}$.** (a)-(b) Landau fan in $\sigma_{xx}$ and $\sigma_{xy}$, respectively. Data measured at $T$ = 1.5 K, and the $D_{eff}$ was varied from -0.6 to 1.1 V/nm, with the $n_{tot}$ fixed at $2.2\times10^{12}$ cm$^{-2}$. Here, $\sigma_{xx}$ and $\sigma_{xy}$ are defined as $\sigma_{xx}=R_{xx}/(R_{xx}^2+R_{xy}^2)$ and $\sigma_{xy}=R_{xy}/(R_{xx}^2+R_{xy}^2)$, respectively. Four-probe lock-in measurements with a frequency of 17.77 Hz were used in this figure.

## 6. Supplementary Note 1. The electrostatic model of the h-BN/Bilayer Graphene (BLG)/CrOCl.

To understand the experimentally observed exotic features, we have in the following developed an electrostatic model in the studied system. We will first list two major unusual experimental observations as follows:

1. The 'iso-doping' line bending effect in the $D_{eff}$-$n_{tot}$ mapping.
2. The large area of gapped state at charge neutrality of BLG, in the $D_{eff}$-$n_{tot}$ mapping.

In the following, we will explain the above two major experimental observations using an electrostatic model. In short, the above observed 'insulating phase edge bending' and the 'huge area of charge neutral state' are attributed to the interfacial coupling between graphene and CrOCl surfaces. Such interfacial coupling invokes two mechanisms:

1) There exist interfacial states, which can serve as a reservoir of electrons with very large density of states (DOS), but have no free charges hence do not contribute to the transport directly.

2) A band structure reconstruction takes place once the Fermi level of graphene matches the lowest energy of the interfacial states, leading to a relative energy shift between interfacial states and BLG

We assume that an interfacial charge layer exists in the h-BN/BLG/CrOCl heterostructure, which is located in bulk CrOCl below graphene with a distance of $d_2$, as shown in Fig. 2c in the main text. The whole system can then be simplified into a capacitance model shown in Fig. 2d in the main text, with charge density defined as $n_t$, $n_1$ (*i.e.*, $n_{BLG}$), $n_2$ (*i.e.*, $n_{CrOCl}$), $n_b$, and the chemical potential defined as $V_{t(g)}$, 0, $V_{b(g)}$. Here, *t* denotes top, *b* denotes bottom. Distance and dielectric constant of each dielectric layer are then written as $d_1$, $\varepsilon_1$, $d_2$, $\varepsilon_1$, $d_3$, $\varepsilon_3$, with the inter-layer electric field written as $E_1$, $E_2$, $E_3$. Moreover, the electron density in graphene and CrOCl layers are defined as $\sigma_1$, and $\sigma_2$, respectively.

Assuming that the interfacial states of BLG/CrOCl are located in the band gap of bulk CrOCl, and with high DOS and width, but do not contribute to transport. When BLG is tuned from the hole-side toward the CNP, *i.e.*, Fermi level ($E_F$) of BLG is lower than the energy of the lowest value of the interfacial states, only charges at the Fermi surface of graphene are at play in the transport. Hence everything is rather conventional, and the system behaves as 'pristine' BLG. The energy difference

between CNP of BLG and the lowest band edge of the interfacial states is defined as $\Delta_E$. It is worth mentioning that the band structure of BLG in our simplified model is parabolic instead of a “W” shape (or, often noted as the ‘Mexican hat’ shape) in reality. However, the modification of band structure has minor effect on the DOS and will not change the main result of our model.

When the BLG band is filled with charge carriers, its $E_F$ increases. At the point when $E_F$ matches the lowest value of the interfacial states, electrons start to fill into the DOS of interfacial states. We will argue in the coming parts that to observe the experimentally observed phenomena, there is an enlargement of $\Delta_E$.

In the coming calculations, DOS for the interfacial states and BLG are written as,

$$\rho_{\mathrm{CrOCl}}(\varepsilon) = \rho \tag{1}$$

$$\rho_{\mathrm{BLG}}(\varepsilon) = \begin{cases} \rho_{\mathrm{Gr}}, E_{\mathrm{F}} > \dfrac{\Delta_{\mathrm{gap}}}{2} \text{ or } E_{\mathrm{F}} < -\dfrac{\Delta_{\mathrm{gap}}}{2} \\ 0, -\dfrac{\Delta_{\mathrm{gap}}}{2} < E_{\mathrm{F}} < \dfrac{\Delta_{\mathrm{gap}}}{2} \end{cases} \tag{2}$$

where we assume that the DOS of BLG is a constant $\rho_{\mathrm{Gr}}$. The band gap $\Delta_{\mathrm{gap}}$ is determined by external electric field $E$, the layer distance $z_0$ of BLG, and the maximum band gap $\gamma_1$ as follows [10]:

$$\Delta_{\mathrm{gap}} = \frac{|U\gamma_1|}{\sqrt{U^2 + \gamma_1^2}} \tag{3}$$

Here

$$U = eEz_0 \tag{4}$$

And $E$ is the average electric field above and below the BLG, $z_0 \sim 0.33$ nm is the layer distance of BLG. We noticed that, experimentally, $d_1/\varepsilon_1$ and $d_3/\varepsilon_3$ can be almost the same, and both $d_1$ and $d_3$ are way larger than $d_2$. For simplicity, we define

$$d_2 = l \tag{5}$$

$$d_1/\varepsilon_1 = d_3/\varepsilon_3 = \alpha l \tag{6}$$

Here, α is a large dimensionless coefficient.

Electron charge is written as $-e$, with $e=1.6\times10^{-19}$ C.

Each electric field can be written as

$$E_1 = \frac{V_t}{d_1} \tag{7}$$

$$E_2 = \frac{V_2}{d_2} \tag{8}$$

$$E_3 = \frac{V_b - V_2}{d_3} \tag{9}$$

Here, we omitted the potential between two layers of BLG since it is usually small compared with the applied voltage on top and bottom gates.

According to the Gauss's law

$$\sigma_1 = -\left(\varepsilon_1 E_1 + \varepsilon_2 E_2\right)\varepsilon_0 = -\frac{(V_t + \alpha V_2)\varepsilon_0}{\alpha l} \tag{10}$$

$$\sigma_2 = \left(\varepsilon_2 E_2 - \varepsilon_3 E_3\right)\varepsilon_0 = \frac{((1+\alpha)V_2 - V_b)\varepsilon_0}{\alpha l} \tag{11}$$

and

$$\sigma_i = -n_i e, i = 1, 2 \tag{12}$$

One obtains the following

$$n_1 = \frac{(V_t + \alpha V_2)\varepsilon_0}{\alpha l e} \tag{13}$$

$$n_2 = \frac{(-(1+\alpha)V_2 + V_b)\varepsilon_0}{\alpha l e} \tag{14}$$

because the chemical potentials of the interfacial states and BLG are equal, one gets

$$n_2 = 0 \tag{15}$$

or

$$n_2 = \rho\left(E_{\mathrm{F}} + eV_2 + \Delta_E\right) \tag{16a}$$

$$n_1 = \begin{cases} \rho_{\mathrm{Gr}}\left(E_{\mathrm{F}} - \frac{\Delta_{\mathrm{gap}}}{2}\right), E_{\mathrm{F}} > \frac{\Delta_{\mathrm{gap}}}{2} \\ 0, -\frac{\Delta_{\mathrm{gap}}}{2} < E_{\mathrm{F}} < \frac{\Delta_{\mathrm{gap}}}{2} \\ \rho_{\mathrm{Gr}}\left(E_{\mathrm{F}} + \frac{\Delta_{\mathrm{gap}}}{2}\right), E_{\mathrm{F}} < -\frac{\Delta_{\mathrm{gap}}}{2} \end{cases} \tag{16b}$$

Here $E_{\mathrm{F}}$ is the Fermi energy. Notice that in the above models, the difference of chemical potential of BLG caused by the movement of its Fermi level is omitted, as it is much smaller compared to the electrostatic energies induced by gate, and does not change the main results.

We then have two phases.:

**Phase-i** (the 'conventional phase' defined in Fig. 2e-f in the main text), $n_2$=0, and the heterostructure act like a "pristine" BLG, and

**Phase-ii** (the 'interfacial coupling phase' defined in Fig. 2e-f in the main text), the

interfacial states are partially occupied.

Since these equations are highly nonlinear, we have to solve them numerically. The parameters used in our calculations are listed below (with S.I. units omitted):

$\alpha = 50$

$l = 4\times10^{-10}$

$\varepsilon_0 = 8.85\times10^{-12}$

$e = 1.602\times10^{-19}$

$\rho = 10^{20}/e$

$\rho_{Gr}=0.5\times10^{18}$

$\Delta_E = 0.26e$

$\hbar = 1.05\times10^{-34}$

$\pi = 3.1415926$

$\gamma_1=0.25e$

Here we derive the DOS of BLG from its electron effective mass which is ~ 0.03 $m_e$, and a coefficient is multiplied to tune the model. In order to have the bending effect of CNP, we assume that $\Delta_E$ is an asymptotic function of $n_2$, which may be a consequence of the bandwidth expansion due to electron interactions in the interfacial states. By taking a hypothesis of a simple asymptotic function, with $A$ and $B$ the fitting parameters:

$$\Delta_E(n_2) = \Delta_E(0) + A\cdot\frac{B\cdot n_2}{\sqrt{(B\cdot n_2)^2+1}} \tag{17}$$

The iso-doping under the assumption of Eq. 17 can be plotted in Fig. 2f in the main text, with $A$ ~ $6\times10^{20}$ and $B$ ~ $1.5\times10^{-17}$ used in the calculations, respectively. It is worth mentioning that without the correction of Eq. 17, the calculated iso-doping lines in phase (ii) will be straight lines with no bending effect.

According to the above analysis, a diagram showing typical transition process in our system from Phase-i to Phase-ii is illustrated in Supplementary Figure 8.

At this stage, our electro-static model well captures the main features of the experimental observation in Fig. 2e in the main text.

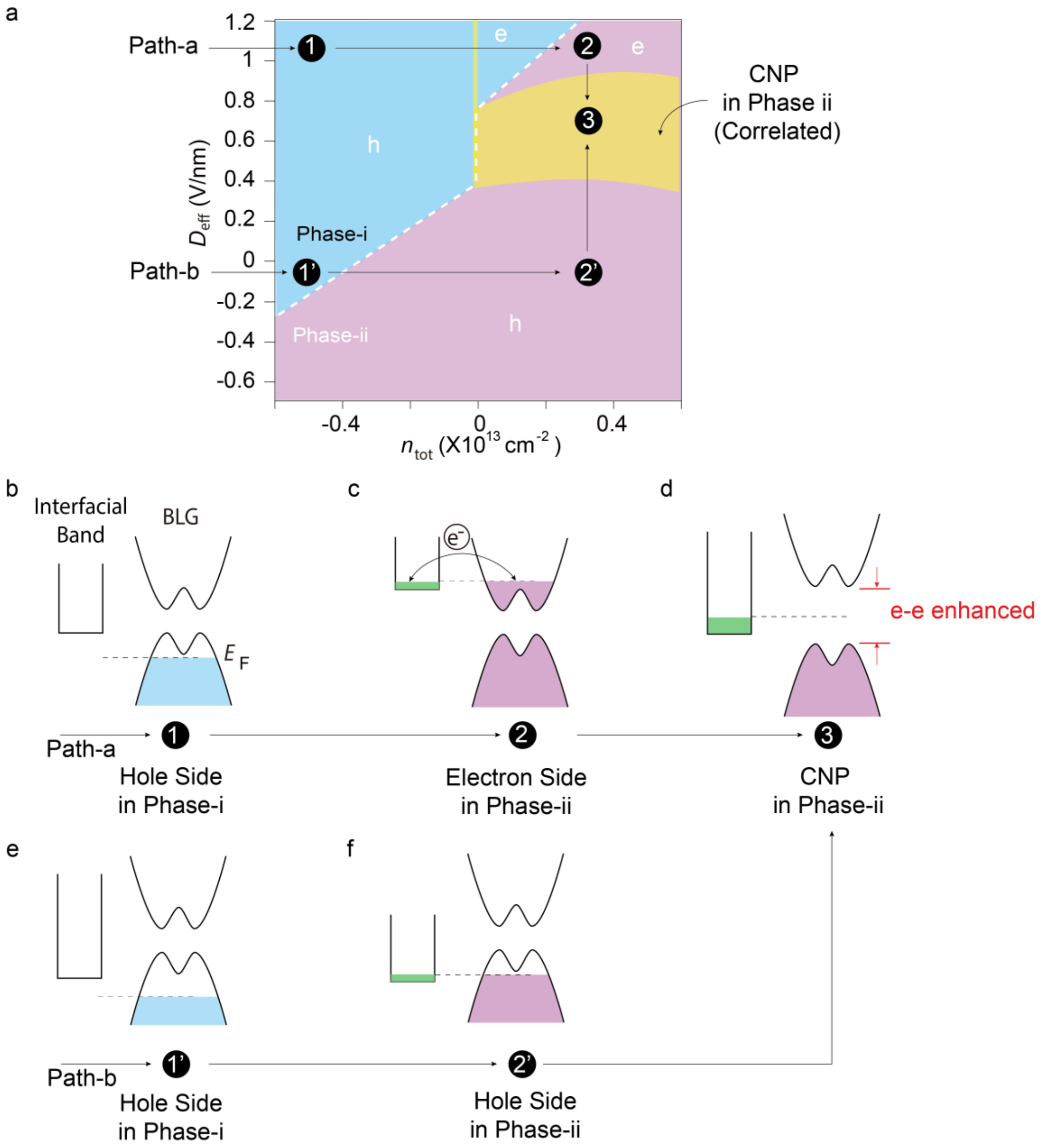


**Supplementary Figure 8.** Schematic of doping-diagram for the transitions between Phase-i and ii.

## 7. Temperature dependence of dual gated $R_{xx}$ in BLG/CrOCl.

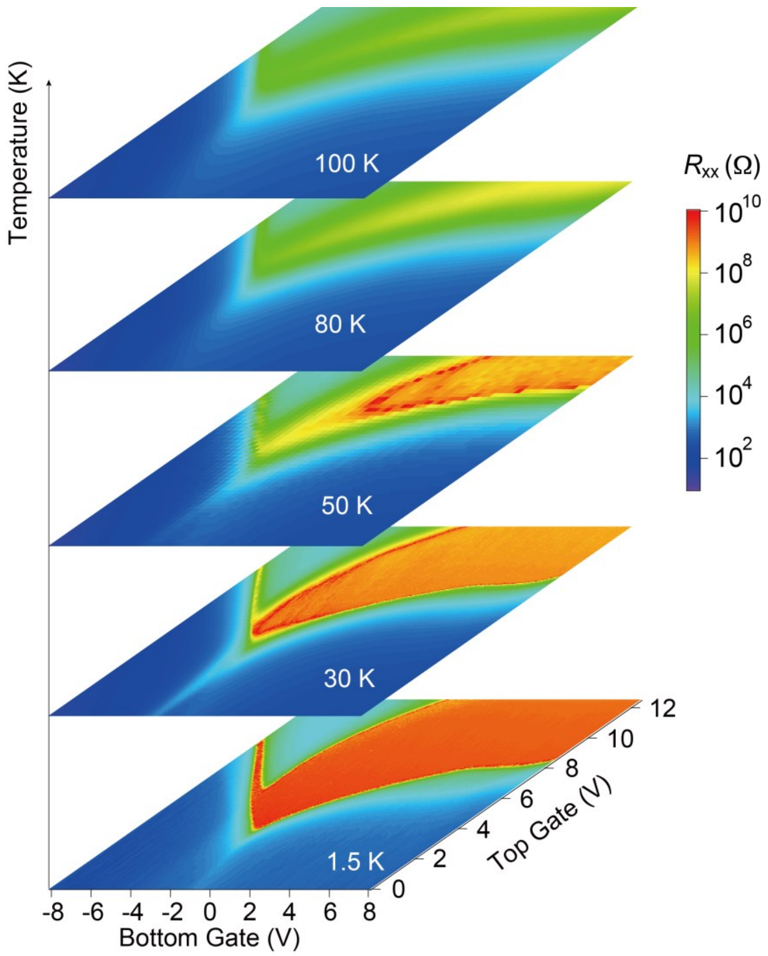


**Supplementary Figure 9**. **Dual gate maps of sample resistance in a typical CrOCl/BLG/h-BN device at different temperatures.** Data were obtained at zero magnetic field, and the temperature was elevated from 1.5 K to 100 K. Two-terminal $R_{xx}$ was recorded using DC measurement. And each map was calibrated by subtracting the contact resistance $R_c$ (measured by four-wire measurement at $V_{tg} = 0$ V and $V_{bg} = -8$ V for each temperature). It is clear that the extremely insulating phase at 1.5 K (orange color, with resistance > 1 GΩ) shrinks its doping range of $D$, along with a decrease of the amplitude of resistance. The insulating phase prevails up to 100 K, but with shrunk phase area and lowered value of resistance.

a

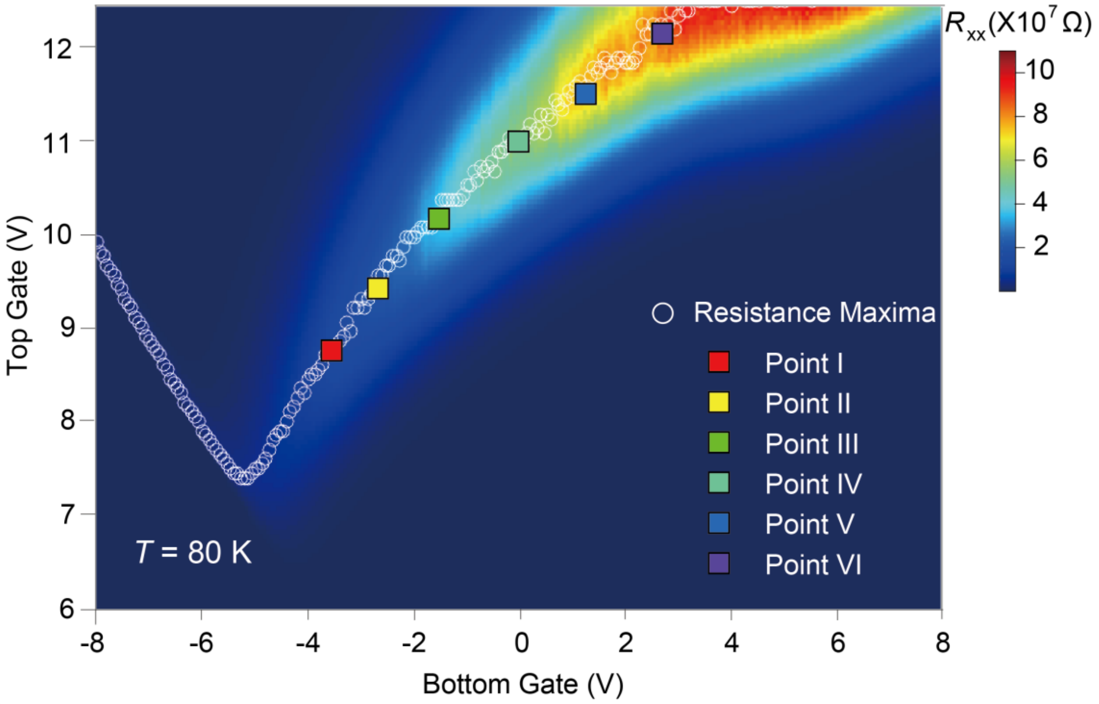


b

| | I | II | III | IV | V | VI |
|---|---|---|---|---|---|---|
| $n_{tot}$ (X10$^{12}$ cm$^{-2}$) | 2.7628 | 4.0438 | 5.8731 | 7.9305 | 9.4372 | 11.310 |
| $D_{eff}$ (V/nm) | 0.8419 | 0.8261 | 0.7944 | 0.7498 | 0.6886 | 0.6276 |

**Supplementary Figure 10. Dual gated map of $R_{xx}$ of a typical BLG/CrOCl sample at $T$ = 80 K.** (a) DC sample resistance measured in a two-wire configuration in the parameter space of $V_{bg}$ and $V_{tg}$ at 80 K. It is seen that the insulating region is much shrunk as compared to that of, for example, $T$ = 1.5 K. Also, the maximum resistance also reduces to a few tens of MΩ, while it reached above 1 GΩ at lower temperatures. Nevertheless, at this temperature (above liquid nitrogen temperature), the insulating phase is still way higher compared to those values reported elsewhere in dual gated bilayer graphene even at temperatures lower than 4 K.[6, 8-9] By extracting the maximums of the resistive peaks, we obtain the white circled line, as indicated in (a). Specifically, we chose 6 points (point-I to VI, indicated in (a)) in the insulating phase to perform thermal activation measurements, as shown in Supplementary Figure 10. (b) Table of the calculated $n_{tot}$ and $D$ for the test-points I-VI using their coordinates of $V_{tg}$ and $V_{bg}$.

## 8. Linear dependence of switching voltage from insulating to metallic state.

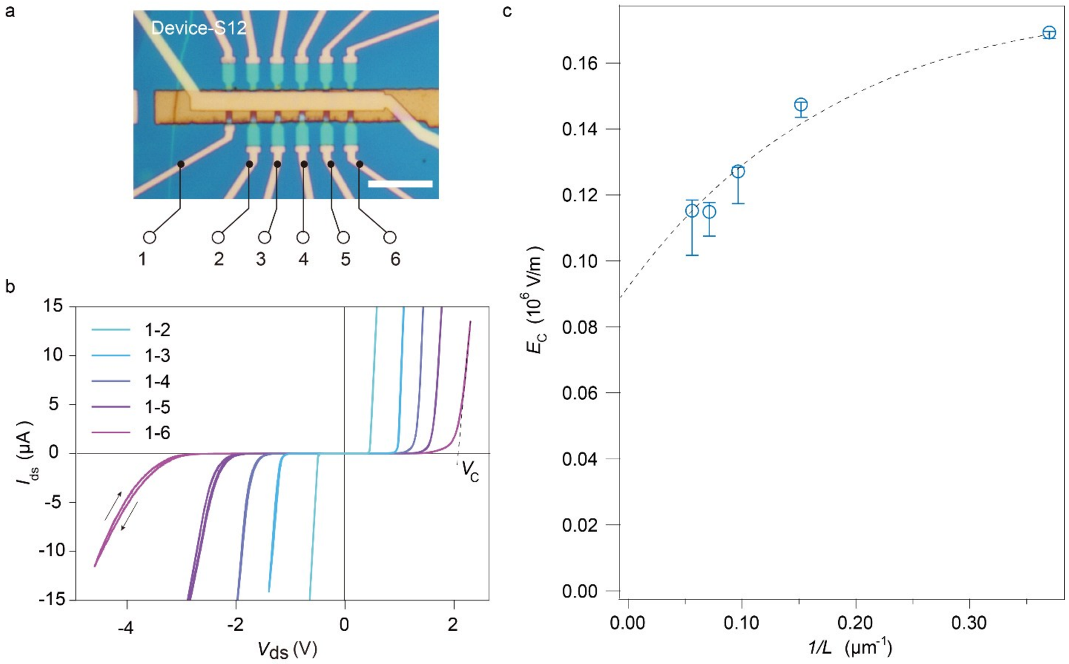


**Supplementary Figure 11. Two-terminal $I_{ds}$-$V_{ds}$ curves measured in electrodes with different distances.** (a) Optical image of a typical sample Device-S12. Scale bar is 10 μm. (b) Two-wire *I-V* curves probed using different pairs of pins, at $T = 1.5$ K and $B = 0$ T, with $D_{eff} \sim 0.4$ V/nm, and $n_{tot} \sim 3\times10^{12}$ cm$^{-2}$. It is clear that the critical voltage (where the system exits the insulating states and enters a resistive 'normal' state, with the relation of *I-V* becoming quasi-linear) is proportional to the distance $L$ of electrodes, between which $V_{ds}$ is applied. Therefore, the critical voltage is actually denoting a critical in-plane electrical field $E_{/\!/}$, defined as $E_{/\!/} = V_{ds}/L$. c) Breakdown in-plane electric field $E_c = V_c/L$, plotted as a function of $1/L$. The fact that the data points do NOT extrapolate to zero is a strong evidence that the current system is NOT of the Zener-type, but rather exhibits a pair-breaking behavior.[11] The dashed black line is guide to the eye. In other words, according to the relationship of $E_c$ vs. $1/L$ in band insulators, the conventional Zener breakdown voltage $E_c = E_c *L$ therefore should have little size-dependence, which is clearly ruled out in our system. It is also worth noting that the mesoscopic samples studied in our work exhibit insulating breakdown at $E_c \sim 10^5$ V/m, orders of magnitudes smaller than the values of Zener breakdown for band insulators ($\sim 10^8$ V/m, which is size-independent).[12] This is in agreement with the theoretical model in in the limit of small $L$.[11]

## 9. Logic devices based on correlated insulator in BLG/CrOCl heterosystem.

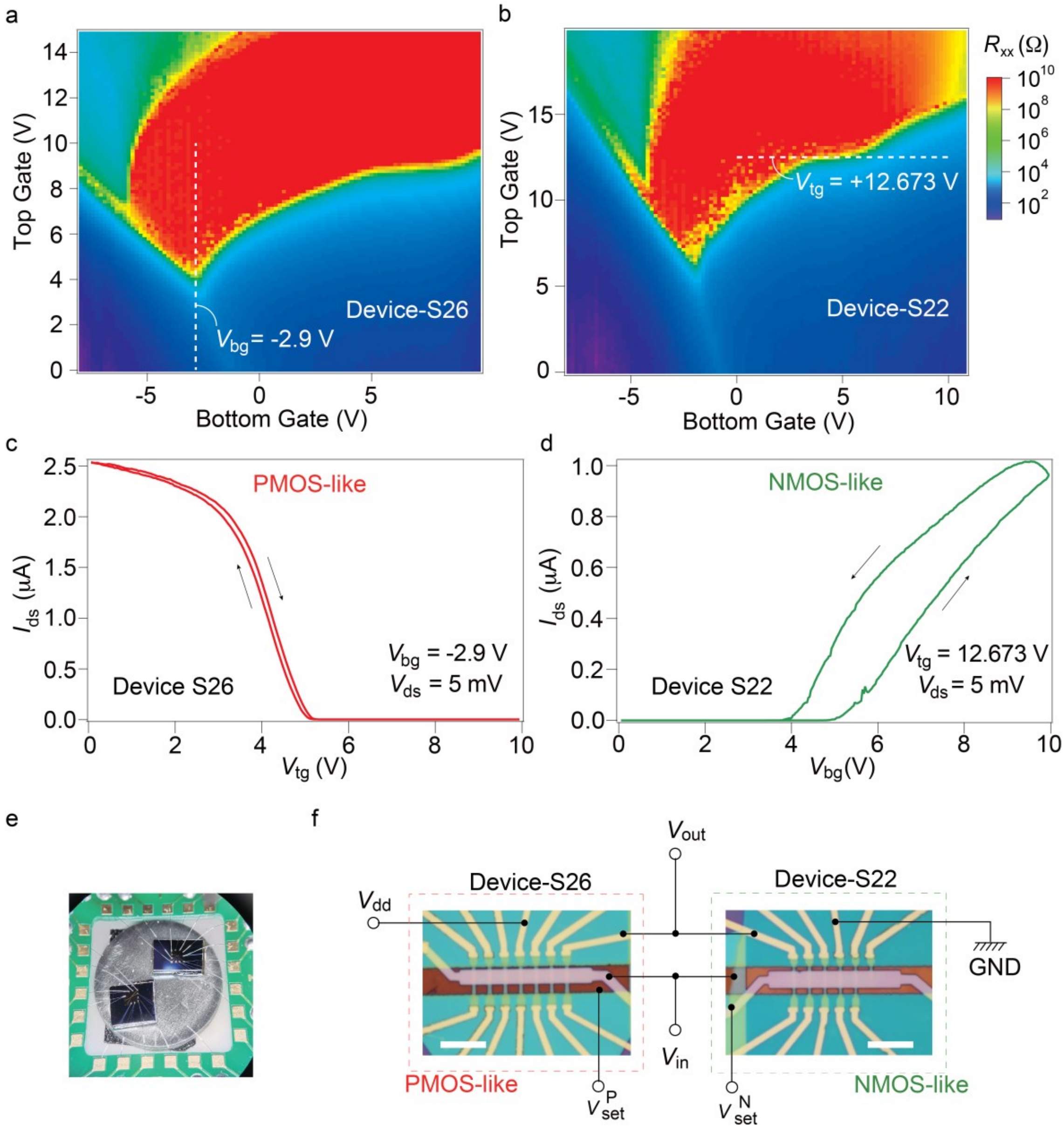


**Supplementary Figure 12. CMOS and logic inverter made of CrOCl-proximitized BLG.** $R_{xx}$ measured at $T$= 1.5 K and $B$=0 T for (a) Device S8 and (b) S7 are shown by sweeping top and bottom gates. Data obtained in a DC mode with a two-wire configuration. The final color map was calibrated by removing the contact resistance of about 1~2 kΩ using four-wire measurement. When the bottom gate (top gate) is set to be constant, the Device S8 (Device S7) can be functioning as a PMOS-like (NMOS-like) transistor by sweeping its top gate (bottom gate) in a voltage range of 0-8 V, shown in (c) and (d), respectively. Here, (c) and (d) are the same data as in Fig. 4a-b in the main text. (e) The optical image of the two devices bonded for measurements. (f) The wiring of the two devices as a logic inverter, as illustrated by the diagram in Fig. 5c in the main text. Scale bars are 5 μm.

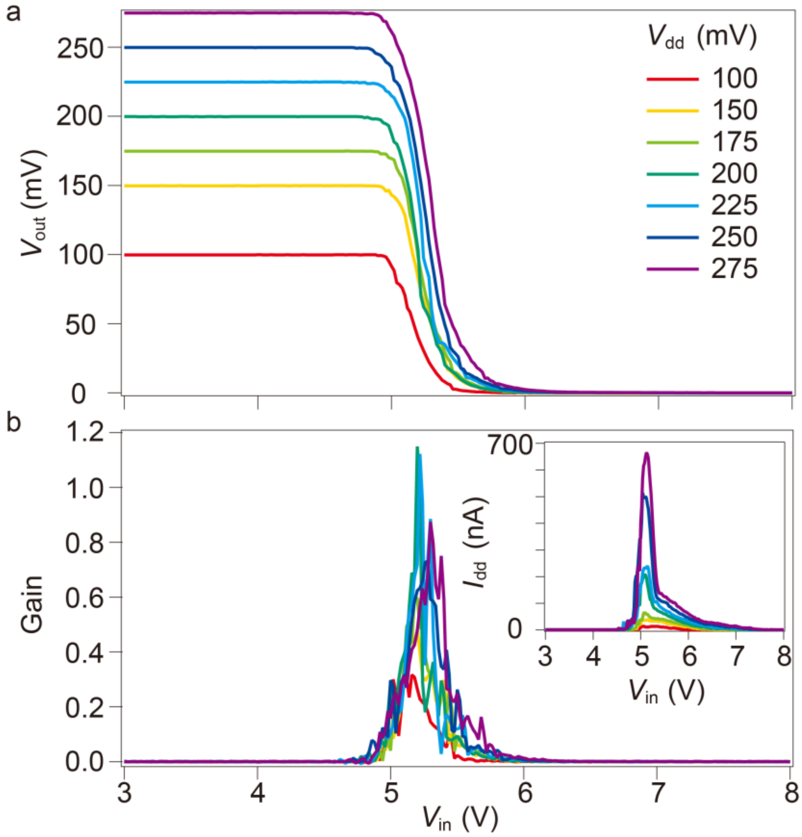


**Supplementary Figure 13**. **Inverter performance at different $V_{dd}$.** (a) $V_{out}$ as a function of $V_{in}$ for different $V_{dd}$, from 100 mV to 275 mV, respectively. (b) The value of gain for each corresponding curves in (a), with $I_{dd}$ (drain current) plotted in the inset.

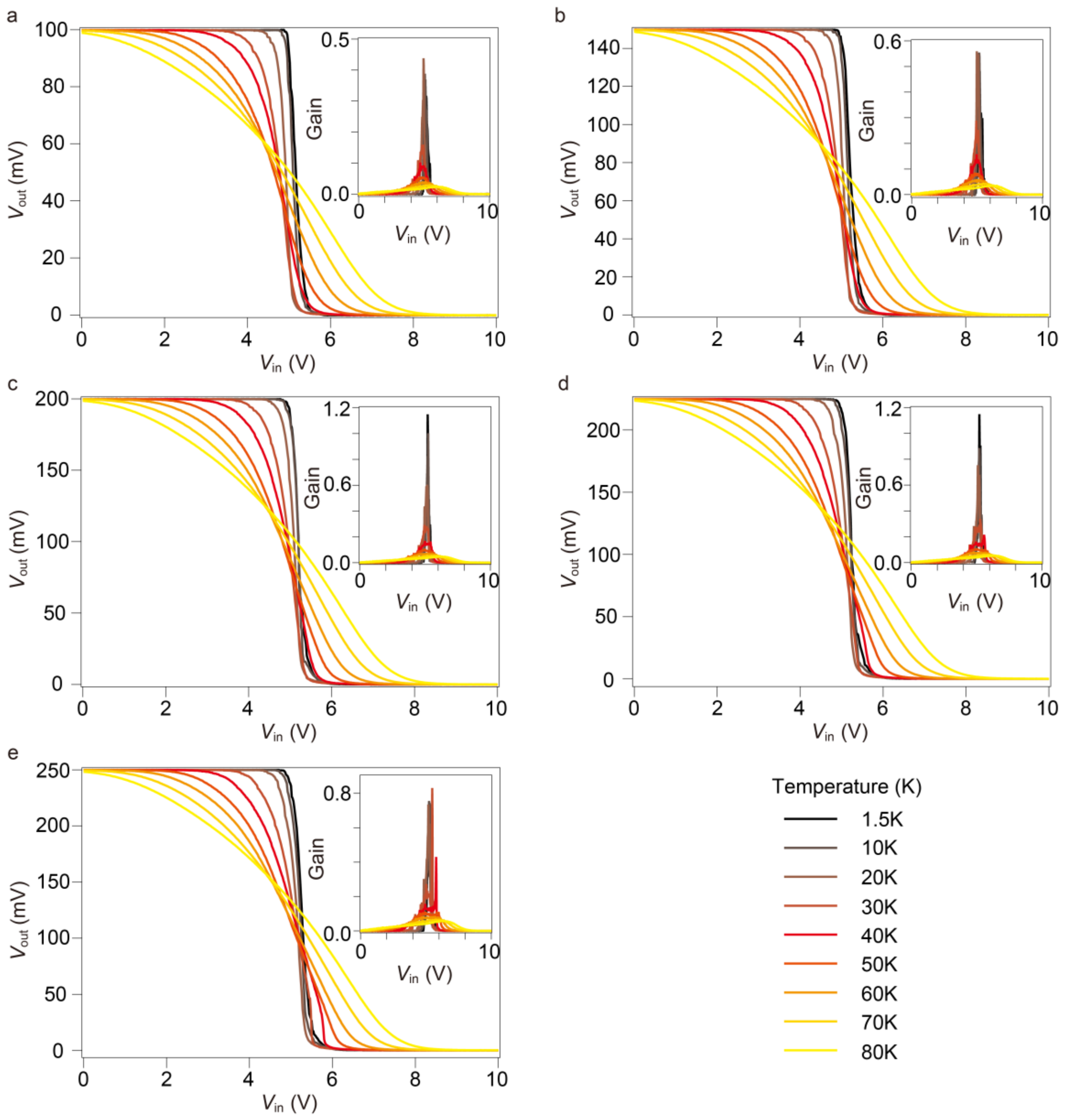


**Supplementary Figure 14**. **Inverter performance at fixed values $V_{dd}$ for different temperatures.** (a)-(e) $V_{out}$ as a function of $V_{in}$ for different temperatures, with the $V_{dd}$ fixed at 100, 150, 200, 225, and 250 mV, respectively. In each sub figure, $V_{out}$ at different temperatures are recorded, from 1.5 K to 80 K. Notice that the (c) here is the same data presented in Fig. 5f in the main text.

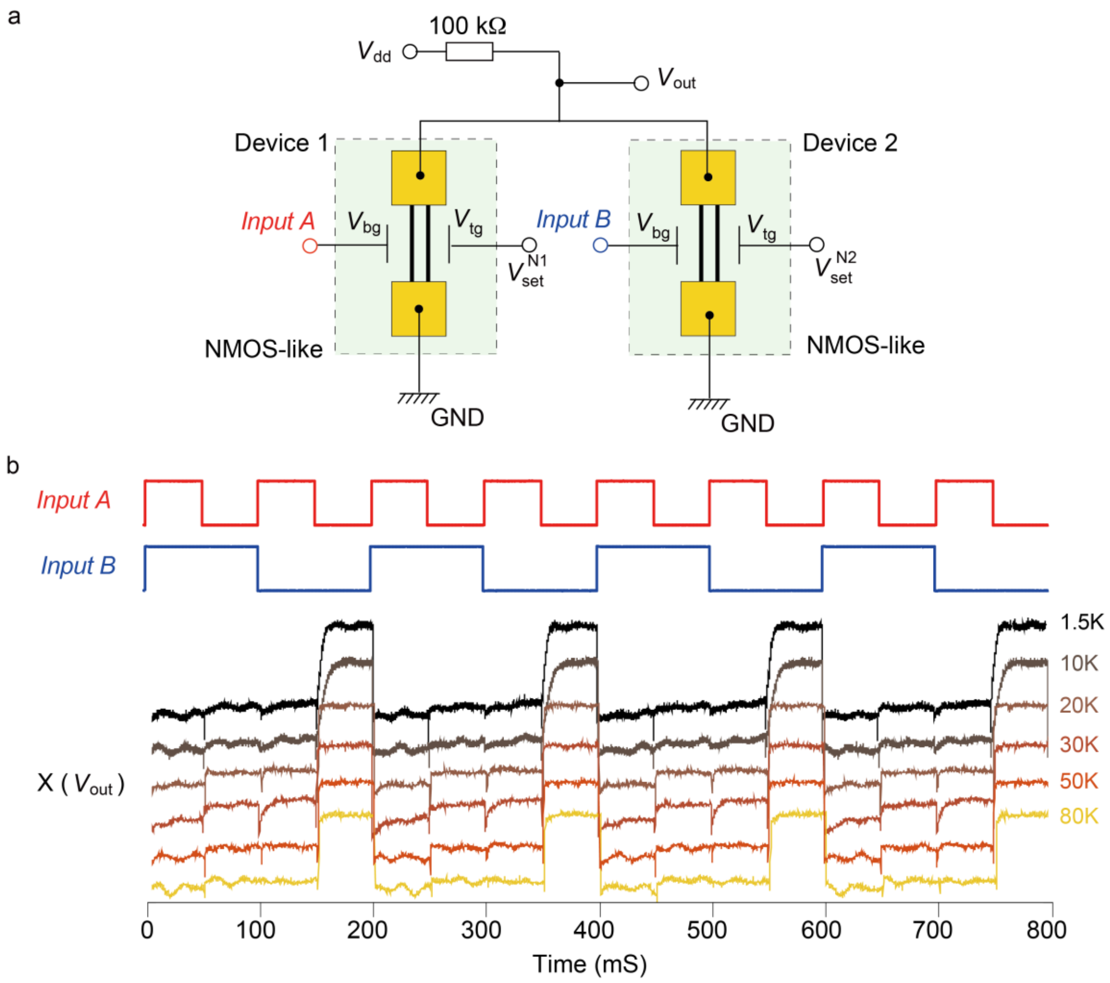


**Supplementary Figure 15**. **A logic NAND gate made of two graphene NMOS-like transistors.** (a) Schematic diagram showing the principle of how the NAND logic gate works with two BLG/CrOCl NMOS-like transistors in parallel. In this particular case, we used Device S22 and Device S26 in parallel, while a $V_{dd}$ is biased through a resistor of 100 kΩ. Notice that both devices are working in the NMOS-like regime, with $V_{tg}$ for Device S22 and Device S26 set to be +7 V and +6 V, respectively. The inputs $A$ and $B$ for Device S22 and Device S26 are given as rectangular waveforms with the frequency of $B$ doubled than that of $A$, and both amplitudes are 5 V. The waveforms of $V_{out}$ ($X$ value in (b)), input $A$, and input $B$ are shown in (b) for different temperatures up to 80 K, which demonstrate a standard logic NAND function.

**Supplementary References**


[1] Wang, L. *et al.*, One-dimensional electrical contact to a two-dimensional material, ***Science***, **342**, 614 (2013).

[2] Lee, K. *et al.*, Chemical potential and quantum Hall ferromagnetism in bilayer graphene, ***Science***, **345**, 58 (2014).

[3] Taychatanapat, T. and Jarillo-Herrero, P., Electronic Transport in Dual-gated Bilayer Graphene at Large Displacement Fields, ***Phys. Rev. Lett.*** **105**, 166601(2010).

[4] Bao, W. *et al.*, Evidence for a spontaneous gapped state in ultraclean bilayer graphene, ***Proc. Natl. Acad. Sci. U. S. A.***, **109**, 10802 (2012).

[5] Zhu, M. *et al.*, Edge currents shunt the insulating bulk in gapped graphene, ***Nat. Commun.***, **8**, 14552 (2017).

[6] Weitz, R. T. *et al.*, Broken-symmetry states in doubly gated suspended bilayer graphene, ***Science*** **330**, 812 (2010).

[7] Maher, P. *et al.*, Tunable fractional quantum Hall phases in bilayer graphene, ***Science*** **345**, 58 (2014).

[8] Zhang, Y. *et al.*, Direct observation of a widely tunable bandgap in bilayer graphene, ***Nature*** **459**, 820 (2009).

[9] Li, J. *et al.*, Effective landau level diagram of bilayer graphene, ***Phys. Rev. Lett.***, **120**, 047701 (2018).

[10] McCann, E., Abergel, D. S., & Fal'ko, V. I., The low energy electronic band structure of bilayer graphene. *The European Physical Journal Special Topics*, **148**, 91-103 (2007).

[11] Shao, Y. & Dai, X., Electrical breakdown of excitonic insulator. arXiv 2302.07543 (2023).

[12] Seabaugh, A. C. and Zhang, Q., Low-Voltage Tunnel Transistors for Beyond CMOS Logic, *Proceedings of the IEEE*, **98**, 2095 (2010).
